# Clay Attenuates Diarrhea Induced by Fat in a Mouse Model

**DOI:** 10.3390/metabo15070483

**Published:** 2025-07-17

**Authors:** Shalom Emmanuel, Nyma Siddiqui, Ting Du, Eric Asare, Yuan Chen, Huan Xie, Dong Liang, Song Gao

**Affiliations:** Department of Pharmaceutical Science, College of Pharmacy and Health Sciences, Texas Southern University, 3100 Cleburne Street, Houston, TX 77004, USA; s.emmanuel2414@student.tsu.edu (S.E.); n.siddiqui6955@student.tsu.edu (N.S.); du.ting@tsu.edu (T.D.); e.asare2673@student.tsu.edu (E.A.); yuan.chen@tsu.edu (Y.C.); huan.xie@tsu.edu (H.X.); dong.liang@tsu.edu (D.L.)

**Keywords:** clay, high-fat diarrhea, fecal bile acid, mouse diarrhea model

## Abstract

**Background:** Diarrhea induced by an excessive amount of fat is a prevalent gastrointestinal disorder. Currently, there are limited animal models and treatment options for diarrhea associated with fat. This study aims to develop a mouse model of high-fat-associated diarrhea using glyceryl-trioleate (GTO) and evaluate the potential of montmorillonite clay (MMT) in mitigating this condition. **Methods:** GTO was administered to mice at different doses through oral gavage to induce diarrhea. Clay was treated through oral gavage to evaluate its anti-diarrhea effect. Fecal conditions were monitored. Intestinal tissues were subjected to histological examination to assess structural integrity. The total fecal bile acids were evaluated using a bile acid assay kit to determine the mechanism of action. **Results:** The results showed that a diarrhea model was established by administering GTO at 2000 mg/kg. When the animals were treated with clay, diarrhea incidence and severity were decreased significantly in a dose-dependent manner. Compared to the untreated group receiving GTO alone, clay co-administration at 2000 mg/kg reduced diarrhea scores by approximately 48%, while the higher dose of 4000 mg/kg achieved an 83% reduction. Fecal bile acid analysis showed that diarrhea is associated with total bile acid levels in the feces. Histological exams showed that diarrhea is associated with tissue inflammation in the colon. **Conclusions:** This study showed that GTO administration induced diarrhea in mice, and clay effectively alleviates fat-induced diarrhea through modulation of fecal bile acid composition. These findings suggest that this model can be used to evaluate diarrhea associated with excessive amounts of fat and clay that can be further tested for diarrhea attenuation.

## 1. Introduction

It is well-known that high-fat diets are usually tasty, but not good for health. However, changing dietary habits away from high-fat diet can be challenging for many people. High fat in diets may lead to GI disorders, including diarrhea, because excessive amount of fat in the diet may cause insufficient fat digestion (lipid metabolism), and non-digested fat could enter the colon, leading to various consequences. For example, it was reported that high-fat feeding can exacerbate colitis in animal models by increasing damage to mucosal tissues and pro-inflammatory cytokines [1]. Epidemiological evidence suggests that populations consuming high fat have a higher prevalence of GI disorders such as celiac disease, IBD, and colorectal cancer [2,3,4]. Therefore, the relationship between non-digested fat and GI disorder should be paid significant attention.

In addition to high-fat diets, non-digested fat-associated GI disorder may also arise from inhibition of pancreatic lipase (PL) in obesity treatment. It was reported that due to PL inhibition, dietary fat could not be fully digested (i.e., metabolized), leading to an accumulation of fat in the GI tract, which can cause symptoms such as fecal spotting, restroom urgency, oily stools, etc. For example, in a large size clinical studies involved in 743 patients, the incidences of fecal urgency, fecal spotting, and oily stool were 7%, 17%, 26% higher, respectively, in the orlistat, an approved PL inhibitor to treat obesity, treatment group when compared to those in the control group [5]. Another clinical trial showed that the incidences of fecal urgency, fecal spotting, and oily stool were 12.5%, 12.5%, and 17.5% higher, respectively, in the treatment group than those in the control group [6]. These results suggested that non-digested fat caused by GI disorder is also a significant side effect in drug development. However, animal models for GI disorders resulting from non-digested fat are understudied, and effective anti-diarrheal approaches have yet to be well developed.

Clays have been historically used for treating diarrhea due to their ability to absorb water, toxins, and lipids in the gut. However, their potential to attenuate fat-induced diarrhea by adsorbing excess fats remains unexplored. This study addresses this critical knowledge gap by (1) developing a reproducible mouse model of diarrhea associated with excessive amounts of fat by oral administration of glyceryl trioleate (GTO), (2) evaluating the therapeutic efficacy of clay in mitigating diarrheal symptoms, and (3) exploring the mechanism of action. We hypothesize that clay (MMT, montmorillonite K10) reduces diarrhea severity via fat adsorption and restoring bile acid balance. The outcomes of this study could provide the scientific rationale for deploying clay-based therapies as safe and effective interventions for managing fat malabsorption-related gastrointestinal disorders.

## 2. Materials and Methods

### 2.1. Chemicals and Reagents

Glyceryl Trioleate (GTO) was purchased from Sigma Aldrich (St. Louis, MO, USA). Clay (montmorillonite K10, MMT) and Total bile acid assay kit (MAK-309) were also purchased from Sigma Aldrich (St. Louis, MO, USA). Deionized water and phosphate-buffered saline (PBS) were purchased from Thermo Fisher Scientific (Waltham, MA, USA). Other chemicals were used as received.

### 2.2. Preparation of Solutions

GTO formulation was prepared by mixing GTO (60% purity, 0.91 g/mL) with Tween 80, Cremophor EL, PEG 400 (1:1:2), and water, according to the guidelines in [7]. MMT clay was prepared in Ora-Plus oral suspending vehicle and vortexed to be used for dosing.

### 2.3. Diarrhea and Anti-Diarrhea by MMT

The diarrhea model was developed by administering GTO through oral gavage. Briefly, animals (adult C57BL/6 mice) were fed with regular diets for at least two weeks after receiving them from vendors. We administered GTO to mice orally at increasing doses of 100 mg/kg/day, 200 mg/kg, 1000 mg/kg, and 2000 mg/kg (n = 10) daily for five days. A washout period was observed between doses. Grade 3 diarrhea was observed from the 2000 mg/kg dose. Thus, we established a diarrhea model at a dose of 2000 mg/kg.

To determine the anti-diarrhea efficacy of clay (MMT), adult C57BL/6 mice were divided into three groups treated with GTO (control group, 2 g/kg/day GTO only), GTO (2 g/kg/day) + low-dose of clay (low dose treatment group, 2 g/kg/day Clay), and GTO (2 g/kg/day) + high dose of clay (high dose treatment group, 4 g/kg/day Clay). Both body weight and fecal conditions were monitored daily to track the index score progression. To ensure consistency, each mouse was placed in a separate cage, and cages were cleaned daily after observation and collection of fecal pellets. Treatment was also administered at the same time each day and placed on a weighing scale for body weight measurement. Furthermore, fecal assessments were performed based on a standardized grading system: grade 1 (wet), grade 2 (soft), and grade 3 (watery), with systematic records noted accordingly. The evaluation of diarrhea scores was carried out continuously from day one to day five to capture the severity and progression of symptoms

### 2.4. Fecal Total Bile Acid Measurement

The total bile acid in the feces was quantified using a Total Bile Acid Assay Kit (MAK-309, Sigma-Aldrich) following the steps in the protocol from the manufacturer. The feces collected on Day 2, one day before the occurrence of severe diarrhea (grade 3), were used for total bile acid determination. Briefly, feces (~500 mg) were weighed accurately in a 2 mL centrifuge tube. Then, 1.0 mL of phosphate-buffer saline (PBS) was added to the tube, followed by homogenization for 1 min and then centrifuged for 15 min at 14,000 rpm under controlled 4 °C temperature. The supernatant was collected and assayed using a 96-well plate and read with a fluorescence reader at 530 nm ex/585 nm em to determine bile acid concentration according to the protocol described in the kit. A standard bile acid mix working solution of 120 μM, containing six bile acids, including Cholic acid (CA), Glycocholic acid (GCA), Taurocholic acid (TCA), Chenodeoxycholic acid (CDCA), Lithocholic acid (LCA), and Deoxycholic acid (DCA), was used as a positive control, while deionized water was used as a negative control.

### 2.5. Histological Study

To examine the effect of GTO on GI tissues, the mice were euthanized at the end of the study, and the colon tissue samples were collected for histological examination. To investigate the effect of GTO on GI tissues, mice were euthanized on the sixth day of therapy, and colon tissue samples from each group were stored in formalin for 24 h. After being gradually dehydrated with ethanol, the samples were embedded in paraffin wax to allow for sectioning. Thin sections, ranging in thickness from 5 to 10 m, were carefully prepared, and the slices were stained with hematoxylin and eosin (HE) for extensive morphological study. It was then examined under a microscope to assess the extent of tissue damage.

The histological exam was graded using the standard reported previously [8,9,10]. Briefly, a score of 0 shows normal histology, whereas as score of 1 shows villus blunting, loss of crypt architecture, sparse inflammatory cell infiltration, vacuolization, and edema in the mucosa and normal muscular layer; a score of 2 shows villus blunting with fattened and vacuolated cells, crypt necrosis, intense inflammatory cell infiltration, vacuolization, and edema in the mucosa; and a score of 3 shows villus blunting with fattened and vacuolated cells, crypt necrosis, intense inflammatory cell infiltration, vacuolization, and edema in the mucosa; and edema, vacuolization, and sparse neutrophil infiltration in the muscular layer.

### 2.6. Statistical Analysis

Statistical variances were analyzed using a *t*-test, with a significance level of *p* < 0.05. GraphPad Prism software (version 7.3 for Windows) was used to perform all statistical analyses. This method ensured an exhaustive analysis of the data, allowing for a complete comparison of the experimental groups.

## 3. Results

### 3.1. Fat-Induced Diarrhea Model in Mice

**Figure 1:**
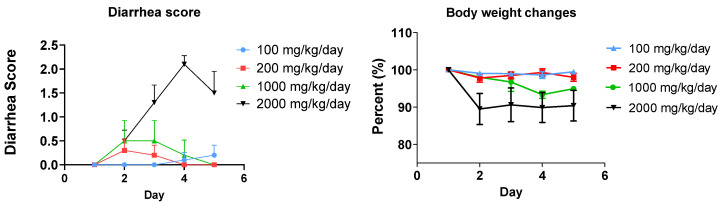
The diarrhea score was evaluated during the five days of treatment. Diarrhea incidence rose dose-dependently at 0.2% at 100 mg/kg, 0.3% at 200 mg/kg, 0.5% at 1000 mg/kg, and 2.1% at 2000 mg/kg of GTO. The results are expressed as mean ± SEM (n = 10).

We initially administered GTO to mice orally at 100 mg/kg/day for 5 consecutive days. Mice did not experience severe diarrhea or any loose stool. Then, the dose was increased to 200 mg/kg/day and 1000 mg/kg/day for 5 days. Mice start with mild diarrhea. When the dose was increased to 2000 mg/kg/day, mice experienced diarrhea on day 2, and then the severity of diarrhea increased on day 3. On days 4 and 5, diarrhea severity is similar to that on day 3. The average diarrhea score increased from 0.5 on Day 2 to 2.1 on Day 4. Thus, we established a diarrhea model at a dose of 2000 mg/kg/day for 5 consecutive days (Figure 1). Body weight showed that with 2000 mg/kg/day, the average body weight decreased significantly since Day 2. This dose equals approximately 10 g of fat for an adult with a 60 kg body weight based on the allometric scaling calculation. The vehicle did not cause any loose stool.

### 3.2. Anti-Diarrhea Efficacy of Clay (MMT)

**Figure 2:**
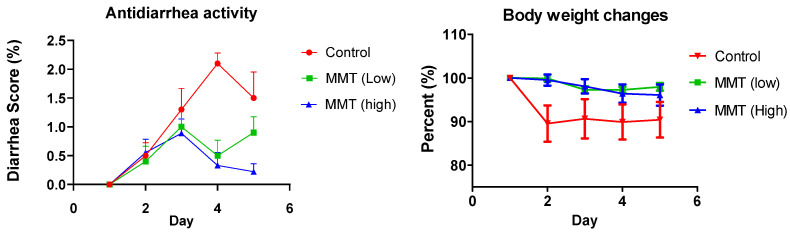
The diarrhea score was evaluated during the five days of treatment. Compared to the control group receiving glyceryl trioleate alone (2000 mg/kg), clay co-administration at 2000 mg/kg reduced diarrhea scores, while the higher dose of clay co-administration at 4000 mg/kg achieved a significant diarrhea attenuation. The results are expressed as mean ± SEM (n = 10).

To evaluate the anti-diarrhea efficacy of clay, the MMT was administered to mice together with GTO using the diarrhea model established above. Two doses of MMT were tested; the results showed that with MMT treatment, diarrhea severity is similar on days 2 and 3 in the control and treatment groups. However, on days 4 and 5, diarrhea severity is significantly lower in the MMT treatment group, suggesting that MMT could treat diarrhea associated with an excessive amount of fat. Interestingly, on Day 5, the anti-diarrhea effect is dose dependent (Figure 2). A high diarrhea incidence of 2.3% was observed in mice treated with GTO alone. However, the diarrhea incidence was reduced by 48% and 83% when clay was co-treated at 2000 mg/kg/day and 4000 mg/kg/day, respectively, and the average diarrhea score decreased significantly from 2.1 to 0.5 and 0.3 on Day 4, respectively (Figure 2). Body weight did not decrease significantly with clay treatment.

### 3.3. Tissue Histological Exam

**Figure 3:**
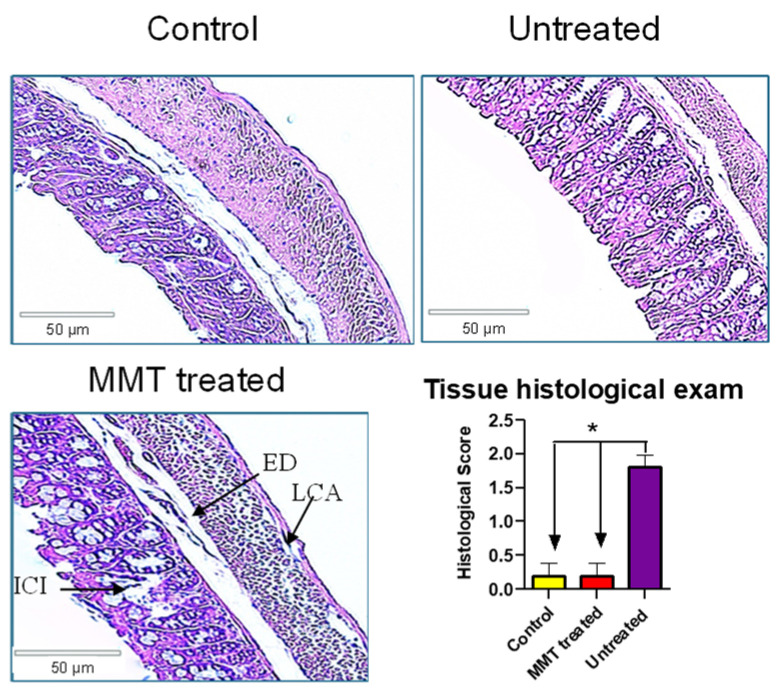
Colon tissue histological exam. Histological sections were stained with H&E (hematoxylin and eosin) and observed microscopically for tissue damage. The control group was treated with vehicle only, the untreated group was treated with GTO (2000 mg/kg/day, p.o.); the MMT-treated group was treated with GTO (2000 mg/kg/day) + MMT 4000 mg/kg/day. The control group shows normal villi, crypts, and a muscular layer. The untreated group shows inflammatory cell infiltration (ICI), loss of normal crypt architecture (LCA), and edema (ED). The MMT-treated group shows restored architecture and reverses the effect of clay damage. Histological score results are expressed as mean ± SEM (n = 5). * *p* < 0.05 (*t*-test).

To further evaluate diarrhea caused by GTO, we collected the colon tissue at the end of the study and did a histological examination. The result showed that compared with the control, the untreated mice had a higher histological score, suggesting tissues were inflamed and damaged with GTO treatment (Figure 3). With MMT treatment, the histological score was decreased to the normal level, suggesting that MMT protects tissues from being inflamed and damaged.

### 3.4. Fecal Bile Acids Level

**Figure 4:**
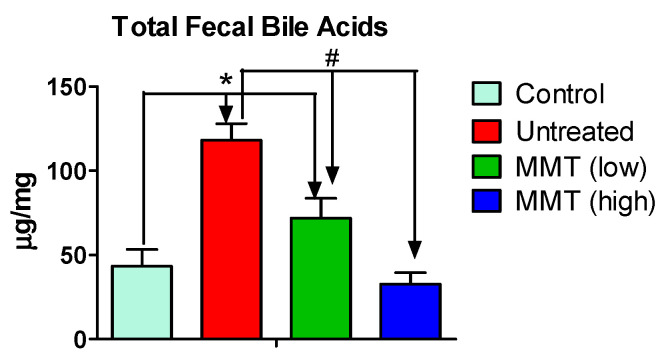
Total bile acid profiling was carried out with the feces of animals from each group on Day 2 (before the occurrence of severe diarrhea), using the Sigma Aldrich bile acid assay kit. The control group received a vehicle only. The untreated group was administered only GTO at 2000 mg/kg/day. The MMT (low) group was administered with 2000 mg/kg/day of MMT and 2000 mg/kg/day of GTO through oral route, and the MMT (high) group was administered with 4000 mg/kg/day of MMT and 2000 mg/kg/day of GTO through oral route. The blank, low-dose, and high-dose groups showed significant differences in the fecal bile acid concentrations compared to the untreated group. Results are expressed as mean ± SEM (n = 5). * *p* < 0.05, comparison with control group, ^#^ *p* < 0.05, comparison with untreated group (*t*-test).

To determine the diarrhea mechanism, we measured the total bile acid in the feces. After clay treatment, fecal bile acid concentrations were significantly lower (*p* < 0.05) than in the untreated group. There was an observed 39.17% decrease in the low-dose group and a 72.31% decrease in the high-dose group. In comparison to the untreated group, the concentration of bile acid was 63.27% lower in the control group, which represented baseline levels, as shown in Figure 4. The results showed that the total bile acids in the feces in the untreated group, which has diarrhea, are significantly higher than those of the control group (2.7-fold), which is only treated with vehicle. Interestingly, in the low and high dose of clay-treated groups, the fecal total bile acids decreased significantly compared to the untreated group. The high clay dose treated group has a similar bile acids level in the feces compared to the control group, while the low dose clay group is slightly higher than the control group (Figure 3) These findings suggested that MMT treatment restore the fecal bile acid levels, which plays a critical role in fat related diarrhea.

## 4. Discussion

In this study, we established a diarrhea model associated with an excessive amount of fat in mice (Figure 1). This model could be used to study high-fat diet-associated GI disorders or GI side effects caused by non-digested fat due to inhibition of pancreatic lipase for obesity treatment. Additionally, we found that clay could significantly mitigate diarrhea associated with non-digested fat (Figure 2). This finding provides a therapeutic option for GI disorders due to fat malabsorption. We also demonstrated that high fat could cause inflammation in the colon (Figure 3), and clay treatment could protect the colon tissue, probably due to absorption of non-digested fat. In terms of mechanism, we found that the total bile acids in the feces were aberrantly elevated with GTO treatment before the occurrence of diarrhea (Figure 4), and clay treatment could significantly restore bile acid levels in the feces, confirming that bile acid plays an essential role in high-fat-associated diarrhea.

High-fat-associated diarrhea and other GI disorders, such as restroom urgency and fecal spotting, are important symptoms in our daily life and in drug development, where targeting inhibition of pancreatic lipase is crucial. However, there is no standard animal model available for therapeutic and mechanistic studies. In this study, we successfully established a reproducible mouse model of fat-induced diarrhea using glyceryl trioleate (GTO), which was evidenced by a dose-dependent increase in the diarrhea severity at increased doses of GTO compared to the baseline (Figure 1). In addition to fecal condition, tissue inflammation status in the colon was also evaluated in this model and the results showed that diarrhea is associated with colonic inflammation (Figure 3). Such an animal model fills a critical gap in preclinical research for high-fat-related GI disorders studies.

Additionally, using this model, we demonstrated that clay (MMT) could significantly mitigate diarrhea associated with high fat (Figure 2) in a dose-dependent manner. Clay has been tested in preclinical and clinical studies to treat diarrhea caused by drugs or diseases. For example, a clinical study showed that clay effectively mitigated diarrhea in medullary thyroid cancer patients [11]. Therefore, it is recognized that clay could effectively mitigate diarrhea [12,13,14]. Mechanisms studies suggested that clay could absorb toxins, water, and proteins to treat diarrhea [15]. In this study, we demonstrated that clay can also treat diarrhea associated with a high-fat diet. It was reported that montmorillonite clay could absorb fat for obesity treatment [15]. In vitro studies showed that montmorillonite can bind up to twice its weight of fat [16]. Therefore, fat absorption by clay is likely to be the major mechanism that mitigates diarrhea in this model.

Mechanism studies have revealed that diarrhea induced by fat is associated with aberrantly elevated bile acids [17]. We observed that in the diarrhea group (i.e., untreated group), the total fecal bile acid level was significantly higher than in the control group, which was treated only with a vehicle, prior to the onset of severe diarrhea (grade 3) on Day 2 (Figure 4). With clay treatment, total bile acids in the feces decreased to normal levels by Day 2, resulting in reduced diarrhea in the following days (Figure 2). It is well-known that fat digestion requires the liver to produce bile, which emulsifies fat and facilitates digestion by pancreatic lipase. However, bile acids could stimulate chloride secretion and disrupt epithelial barrier integrity and accelerate colonic transit, leading to diarrhea. Therefore, it is reasonable to propose that clay absorbs non-digested fat, resulting in reduced bile secretion and alleviated diarrhea.

Our results have potential translational relevance. The dose of 2000 mg/kg GTO in mice approximates a human equivalent dose of approximately 163 mg/kg (9.7 g for a 60 kg adult) based on the allometric scaling guidelines [18]. This dose in mice may not be able to translate directly into humans due to species differences. However, this model demonstrates that excessive amounts of fat result in diarrhea, which is a symptom observed frequently in humans, particularly in patients treated with lipase inhibitors (e.g., orlistat) or those with bile acid diarrhea (BAM) [19].

Using the physical absorption property of clay for diarrhea treatment has been attempted in clinics. For example, beidellitic montmorillonite has shown clinical benefit in a randomized, double-blind, placebo-controlled trial for irritable bowel syndrome (IBS), with 49.4% of patients experiencing significant symptom relief compared to 31.5% in the placebo group [20]. Another example showed that in pediatric patients with acute diarrhea those treated with montmorillonite combined with vitamin AD and zinc supplementation had a significantly higher efficacy rate (94.87%) compared to montmorillonite monotherapy (56.41%), along with faster symptom relief, reduced inflammatory markers (CRP, TNF-α, NO, PCT), and a lower recurrence rate [21]. These findings collectively highlight montmorillonite’s therapeutic potential as a broadly applicable, well-tolerated, and non-pharmacological agent for managing diarrhea associated with diverse etiologies, including fat malabsorption and bile acid dysregulation.

Future studies could focus on exploring biological mechanisms through which MMT modulates bile acid homeostasis. Additionally, bile acid composition analysis using LC-MS is also suggested to map the specific bile acid species most elevated in fat-induced diarrhea (e.g., DCA, LCA, TCA). Such analyses could help target interventions toward the most pathogenic bile acids.

## 5. Conclusions

A fat-associated diarrhea model has been successfully established by orally administering GTO at 2000 mg/kg in mice. In this model, mild diarrhea (grades 1 and 2) begins on Day 2, gradually increasing from Day 3 to Day 5, with severe diarrhea (grade 3) observed on Days 4 and 5, accompanied by tissue inflammation in the colon. The mechanism behind fat-associated diarrhea appears to be aberrantly elevated bile secretion, as total fecal bile acid levels increased approximately 2.7-fold in the GTO-treated group compared to the control group. This model can be utilized to study the mechanisms of fat-associated diarrhea and to evaluate treatments for non-infectious diarrhea. Additionally, our findings suggest that clay effectively reduces fat-associated diarrhea in a dose-dependent manner, achieving a 48% reduction in diarrhea incidence at a low dose (2000 mg/kg) and an 83% reduction at a high dose (4000 mg/kg). These results indicate the potential for further studies to explore clay as an anti-diarrheal treatment in clinical settings.

## Data Availability

All the data are stored in the Research Infrastructure Core (RIC) under the Center for Biomedical and Minority Health Research (CBMHR) at TSU. Data is available for research purposes upon request.

## References

[1] Duan Y., Zeng L., Zheng C., Song B., Li F., Kong X., Xu K. (2018). Inflammatory Links Between High Fat Diets and Diseases. Front. Immunol..

[2] Owczarek D., Rodacki T., Domagala-Rodacka R., Cibor D., Mach T. (2016). Diet and nutritional factors in inflammatory bowel diseases. World J. Gastroenterol..

[3] Cardo A., Churruca I., Lasa A., Navarro V., Vazquez-Polo M., Perez-Junkera G., Larretxi I. (2021). Nutritional Imbalances in Adult Celiac Patients Following a Gluten-Free Diet. Nutrients.

[4] Karunanithi S., Levi L. (2018). High-fat diet and colorectal cancer: Myths and facts. Future Oncol..

[5] Sjostrom L., Rissanen A., Andersen T., Boldrin M., Golay A., Koppeschaar H.P., Krempf M. (1998). Randomised placebo-controlled trial of orlistat for weight loss and prevention of weight regain in obese patients. Eur. Multicentre Orlistat Study Group. Lancet..

[6] Feng X., Lin Y., Zhuo S., Dong Z., Shao C., Ye J., Zhong B. (2023). Treatment of obesity and metabolic-associated fatty liver disease with a diet or orlistat: A randomized controlled trial. Am. J. Clin. Nutr..

[7] Gad S.C., Spainhour C.B., Shoemake C., Pallman D.R., Stricker-Krongrad A., Downing P.A., Seals R.E., Eagle L.A., Polhamus K., Daly J. (2016). Tolerable Levels of Nonclinical Vehicles and Formulations Used in Studies by Multiple Routes in Multiple Species With Notes on Methods to Improve Utility. Int. J. Toxicol..

[8] Geboes K., Riddell R., Ost A., Jensfelt B., Persson T., Lofberg R. (2000). A reproducible grading scale for histological assessment of inflammation in ulcerative colitis. Gut.

[9] Vespa E., D’Amico F., Sollai M., Allocca M., Furfaro F., Zilli A., Dal Buono A., Gabbiadini R., Danese S., Fiorino G. (2022). Histological Scores in Patients with Inflammatory Bowel Diseases: The State of the Art. J. Clin. Med..

[10] Fabian O., Bajer L. (2022). Histopathological assessment of the microscopic activity in inflammatory bowel diseases: What are we looking for?. World J. Gastroenterol..

[11] Dadu R., Hu M.I., Cleeland C., Busaidy N.L., Habra M., Waguespack S.G., Sherman S.I., Ying A., Fox P., Cabanillas M.E. (2015). Efficacy of the Natural Clay, Calcium Aluminosilicate Anti-Diarrheal, in Reducing Medullary Thyroid Cancer-Related Diarrhea and Its Effects on Quality of Life: A Pilot Study. Thyroid.

[12] Guarino A., Lo Vecchio A., Pirozzi M.R. (2009). Clinical role of diosmectite in the management of diarrhea. Expert. Opin. Drug Metab. Toxicol..

[13] Perez-Gaxiola G., Cuello-Garcia C.A., Florez I.D., Perez-Pico V.M. (2018). Smectite for acute infectious diarrhoea in children. Cochrane Database Syst. Rev..

[14] Madkour A.A., Madina E.M., el-Azzouni O.E., Amer M.A., el-Walili T.M., Abbass T. (1993). Smectite in acute diarrhea in children: A double-blind placebo-controlled clinical trial. J. Pediatr. Gastroenterol. Nutr..

[15] Garcia-Garcia F.A., Cristiani-Urbina E., Morales-Barrera L., Rodriguez-Pena O.N., Hernandez-Portilla L.B., Flores-Ortiz C.M. (2023). Spectroscopic and Microestructural Evidence for T-2 Toxin Adsorption Mechanism by Natural Bentonite Modified with Organic Cations. Toxins.

[16] Xu P., Dai S., Wang J., Zhang J., Liu J., Wang F., Zhai Y. (2016). Preventive obesity agent montmorillonite adsorbs dietary lipids and enhances lipid excretion from the digestive tract. Sci. Rep..

[17] Appleby R., Moghul I., Khan S., Yee M., Manousou P., Neal T.D., Waters J.R.F. (2019). Non-alcoholic fatty liver disease is associated with dysregulated bile acid synthesis and diarrhea: A prospective observational study. PLoS ONE.

[18] Reagan-Shaw S., Nihal M., Ahmad N. (2008). Dose translation from animal to human studies revisited. FASEB J. Off. Publ. Fed. Am. Soc. Exp. Biol..

[19] Camilleri M. (2015). Bile Acid diarrhea: Prevalence, pathogenesis, and therapy. Gut Liver.

[20] Ducrotte P., Dapoigny M., Bonaz B., Siproudhis L. (2005). Symptomatic efficacy of beidellite montmorillonite in irritable bowel syndrome: A randomized, controlled trial. Aliment. Pharmacol. Ther..

[21] Zhou H., Wang S., Wang L. (2021). Efficacy of montmorillonite and vitamin A combined with zinc preparation in children with diarrheal disease and its effect on inflammatory factors. Am. J. Transl. Res..

